# TOTEM: a multi-cancer detection and localization approach using circulating tumor DNA methylation markers

**DOI:** 10.1186/s12885-024-12626-7

**Published:** 2024-07-15

**Authors:** Dalin Xiong, Tiancheng Han, Yulong Li, Yuanyuan Hong, Suxing Li, Xi Li, Wenhui Tao, Yu S. Huang, Weizhi Chen, Chunguang Li

**Affiliations:** 1 Department of Thoracic Surgery, Yan’an Hospital of Kunming Medical University, Kunming, 650051 China; 2 Genecast Biotechnology Co., Ltd., Wuxi, Jiangsu 214105 China; 3 Department of Gastroenterology, Zhongnan Hospital of Wuhan University, Wuhan, 430071 China; 4 Department of Colorectal and Anal Surgery/Hubei Key Laboratory of Intestinal and Colorectal Diseases, Zhongnan Hospital of Wuhan University, Wuhan, 430071 China; 5 Clinical Center of Intestinal and Colorectal Diseases of Hubei Province, Wuhan, 430071 China; 6 Quality Control Center of Colorectal and Anal Surgery of Health Commission of Hubei Province, Wuhan, 430071 China

**Keywords:** Multi-cancer early detection, cfDNA methylation, Tumor origin

## Abstract

**Background:**

Detection of cancer and identification of tumor origin at an early stage improve the survival and prognosis of patients. Herein, we proposed a plasma cfDNA-based approach called TOTEM to detect and trace the cancer signal origin (CSO) through methylation markers.

**Methods:**

We performed enzymatic conversion-based targeted methylation sequencing on plasma cfDNA samples collected from a clinical cohort of 500 healthy controls and 733 cancer patients with seven types of cancer (breast, colorectum, esophagus, stomach, liver, lung, and pancreas) and randomly divided these samples into a training cohort and a testing cohort. An independent validation cohort of 143 healthy controls, 79 liver cancer patients and 100 stomach cancer patients were recruited to validate the generalizability of our approach.

**Results:**

A total of 57 multi-cancer diagnostic markers and 873 CSO markers were selected for model development. The binary diagnostic model achieved an area under the curve (AUC) of 0.907, 0.908 and 0.868 in the training, testing and independent validation cohorts, respectively. With a training specificity of 98%, the specificities in the testing and independent validation cohorts were 100% and 98.6%, respectively. Overall sensitivity across all cancer stages was 65.5%, 67.3% and 55.9% in the training, testing and independent validation cohorts, respectively. Early-stage (I and II) sensitivity was 50.3% and 45.7% in the training and testing cohorts, respectively. For cancer patients correctly identified by the binary classifier, the top 1 and top 2 CSO accuracies were 77.7% and 86.5% in the testing cohort (*n* = 148) and 76.0% and 84.0% in the independent validation cohort (*n* = 100). Notably, performance was maintained with only 21 diagnostic and 214 CSO markers, achieving a training AUC of 0.865, a testing AUC of 0.866, and an integrated top 2 accuracy of 83.1% in the testing cohort.

**Conclusions:**

TOTEM demonstrates promising potential for accurate multi-cancer detection and localization by profiling plasma methylation markers. The real-world clinical performance of our approach needs to be investigated in a much larger prospective cohort.

**Supplementary Information:**

The online version contains supplementary material available at 10.1186/s12885-024-12626-7.

## Background

Detecting and diagnosing cancer early offers the opportunity for more effective therapeutic interventions to improve patient outcomes [1]. For the purpose of population-scale cancer screening, a minimally invasive test with affordable cost and superior accuracy for multi-cancer detection and localization is urgently needed, especially for cancers without effective screening paradigms. Existing noninvasive blood-based tests based on protein biomarkers cannot achieve satisfactory sensitivity or specificity to direct diagnosis [2]. Nor can they provide clues about the cancer signal origin (CSO). There are huge unmet needs for the search of better biomarkers for blood-based cancer detection.

In recent years, the potential of using genetic or epigenetic aberrations of plasma cell-free DNA (cfDNA) to detect the presence of circulating tumor DNA (ctDNA) has attracted much attention in the field of cancer early detection [3–6]. Among the different types of cfDNA-bearing cancer features, DNA methylation has shown the most promising results for both early detection and CSO localization of multiple cancers [3, 5, 7, 8], probably due to the pervasive nature of cancer genome methylation alterations and the tissue specificity of methylation signatures [9, 10]. Technically, the conventional whole-genome bisulfite sequencing (WGBS) assay can provide a comprehensive methylation profile of the entire genome [11, 12]. However, the large size of the human genome as well as the sparsity of CpGs reduces the cost-effectiveness of WGBS. To increase the sequencing depth for more accurate methylation profiling of characteristic CpG sites, targeted methylation sequencing with capture panels has been employed and validated for multi-cancer detection [3, 13]. However, these previously published panels are relatively large, with their costs still being an obstacle for large-scale screening. Furthermore, these assays relied on bisulfite conversion leading to dramatic DNA degradation [14]. As a result, there are concerns regarding their applications to ctDNA due to the extremely low concentration of ctDNA in the blood of patients with early-stage or difficult-to-shed tumors.

To circumvent the limitations of bisulfite sequencing, a mild enzyme-mediated assay, enzymatic methyl sequencing (EM-seq), has recently been developed. It uses a combination of enzymes, including TET2, T4-βGT, and APOBEC3A to convert unmethylated cytosine (C) to thymine (T) with efficiency comparable to bisulfite conversion and minimal damages to DNA [15]. As a result, EM-seq is expected to increase the recovery rate of valuable cfDNA and improve the efficiency of library construction. The EM-seq conversion reaction is more sophisticated than bisulfite conversion, thus demanding strict adherence to the protocol and a longer experiment time. Despite the higher cost in terms of money and labor, EM-seq experiences fewer under/over-conversion problems and yields higher libraries using fewer PCR cycles for all DNA input than bisulfite conversion [15]. To take advantage of this, a hepatocellular carcinoma detection panel using EM-seq of plasma cfDNA was investigated and showed promising performance [16]. For effective cancer screening in the general population, we propose an EM-seq-based targeted methylation sequencing platform with integrated quality control for multi-cancer detection and subsequent CSO localization to inform diagnostic workup.

In this study, we proposed a novel approach called TOTEM (cTdna Origin Tracker dependent on Epigenetic Methylation markers) to detect and track the origin of ctDNA. A targeted methylation panel, spanning ~ 1 Mb genomic regions and covering 82,400 CpG sites, was designed (Additional file 1: Methods) and validated for the detection and localization of seven common cancers (lung, colorectal, stomach, liver, breast, esophageal and pancreatic) at both early and late stages. These cancer types are the top seven cancers with the highest combined mortality rates in both gender, estimated to contribute to 60.4% of the 9.6 million global cancer deaths in 2018 [17]. This statistic underscores the critical need for early diagnosis in these cancer types. Hypermethylated CpG sites in tumors versus adjacent normal tissues and versus peripheral blood were identified using tissues from The Cancer Genome Atlas (TCGA) database and peripheral blood samples from publicly available data. Using EM-seq, we performed targeted methylation sequencing of plasma cfDNA samples from a clinical cohort of 733 cancer patients and 500 healthy controls, which were randomly divided into a training set and a testing set at a 7:3 ratio. We developed computational methods to profile methylation status at the fragment level, which were used to select and model 57 and 873 methylation markers for cancer diagnosis and localization, respectively. TOTEM demonstrated robust performance, achieving an overall area under the curve (AUC) of 0.908 (95% confidence interval (CI): 0.879–0.938) for cancer detection and an overall top 1 accuracy of 77.7% (95% CI: 70.1%-84.1%) and an overall top 2 accuracy of 86.5% (95% CI: 79.9%-91.5%) for CSO prediction in the test set. Notably, the number of diagnostic markers could be reduced to as few as 21 while maintaining comparable detection power, opening the potential for development of a multiplexed PCR assay for improved affordability and faster turnaround time. Similarly, the number of CSO markers could be reduced to 214, a significant reduction from previously published studies [3, 13].

## Methods

### Study design and participants

To develop and validate TOTEM, we applied the enzymatic conversion-based targeted methylation sequencing to plasma cfDNA samples collected from a clinical cohort of 500 healthy controls and 733 previously untreated cancer patients across the seven highest mortality cancers [17]. These samples were randomly divided into a training cohort and a testing cohort in a 7:3 ratio, stratified by cancer type and stage. The training cohort included 350 healthy controls and 513 cancer patients (39 breast cancer (BRCA), 72 colorectal cancer (COREAD), 75 esophageal cancer (ESCA), 73 liver cancer (LIHC), 106 lung cancer (NSCLC), 78 pancreatic cancer (PAAD), and 70 stomach cancer (STAD) patients), with which we performed marker selection and model construction. The testing cohort consisted of 150 healthy controls and 220 cancer patients (17 BRCA, 31 COREAD, 32 ESCA, 31 LIHC, 47 NSCLC, 33 PAAD, and 29 STAD) for model evaluation. Specifically, 36% (187/513) of the training cancer samples and 37% (81/220) of the testing cancer samples were at early stage (I or II). In addition, an independent validation cohort of 143 healthy controls, 79 LIHC, and 100 STAD patients was recruited to validate the generalizability of TOTEM, after models developed from the training cohort were locked. Funding and time constraints limited our ability to include other cancer types, thus restricting validation to LIHC and STAD. Healthy controls were defined as individuals with no history or presence of cancer at the time of administration. Blood samples from cancer patients were collected prior to surgical or therapeutic treatment. The demographics and clinical information of the participants are summarized (Additional file 1: Table S1).

All participants were enrolled from Zhongnan Hospital of Wuhan University and Yan'an Hospital of Kunming Medical University. This study was approved by the institutional medical ethics committee (Zhongnan Hospital: 2020106, and Yan'an Hospital: 2023–059-01). Prior to enrollment, all participants provided informed consent for research use.

### Plasma cfDNA extraction

A total of ~ 10 ml peripheral blood was drawn and stored in Cell-Free DNA Storage Tube (Cwbiotech). Blood was centrifuged at 1,600 g for 10 min at 4 °C and plasma was transferred to a new tube. A second centrifuge was performed at 12,000 rpm for 15 min at 4 °C and ~ 4 ml of plasma was isolated. cfDNA was extracted using MagMAX Cell-Free DNA Isolation Kit (Thermo Fisher Scientific) per manufacturer instructions. The quantity and quality of extracted cfDNA was assessed with Bioanalyzer 2100 (Agilent).

### Sequencing library preparation

A range of 5 to 30 ng of cfDNA was enzymatically converted with NEBNext Enzymatic Methyl-seq Kit (New England Biolabs) and captured with hybridization probes (Roche Diagnostics) according to manufacturer's protocols. Libraries were quantified using Qubit dsDNA HS Assay Kit (Thermo Fisher Scientific) and sequenced on NovaSeq 6000 (Illumina) with a paired-end read length of 150 bp.

### Panel design and identification of methylation-correlated blocks

To construct a targeted methylation panel for multi-cancer detection and CSO localization, we selected differentially methylated regions (DMRs) using 450 k methylation array data from the TCGA database and a previously published normal peripheral blood dataset (GSE40279 [18]) (details in Additional file 1: Methods). Due to the processivity of DNA methyltransferases, adjacent CpGs with highly similar methylation status were grouped into methylated correlation blocks (MCBs) as the basic unit of methylation markers to reduce technical noise [19] with the following criteria: (1) the distance between the two CpG sites was less than 100 bp; (2) the Pearson's correlation coefficient between the two sites was greater than 0.95; (3) at least three consecutive CpG sites could be combined. The correlation was calculated from the beta-value of 130 tumor tissue samples from various cancer types.

### Multi-cancer diagnostic marker selection

Read pairs covering at least three CpG sites were merged into fragments for methylation analysis. The methylation level of an MCB was quantified by methylated fragment counts (MFCs), which was defined as the number of fragments with a fully methylated status on the MCB. Hypermethylated diagnostic MCB markers of a specific cancer type were selected by mutual information (MI), which assessed the difference in MFC values between the cancer group and the healthy group in the training cohort (Additional file 1: Methods). The MCBs were ranked according to the cancer-specific MI in descending order and the top *X* MCBs were selected as hypermethylated diagnostic markers of a specific cancer type. The union of markers for the seven cancer types was used as the set of multi-cancer diagnostic markers. *X* was optimized by maximizing the diagnostic AUC of the training cohort.

### Diagnostic methylation score model

A metric termed methylation score was constructed to score the abnormality of the methylation pattern between the tested sample and the baseline distribution of healthy controls in the training cohort [20]. To reduce technical noise, the methylation level of an MCB was calculated with the methylated fragment ratio (MFR), which was defined as the percentage of fully methylated fragments.

First, logit transformation was performed on the MFR values prior to modeling. The difference of the logit-transformed MFR ${x}_{i}$ of MCB marker $i$ between the tested sample and the baseline distribution was measured by Z-score.

1 $${Z}_{i}=\frac{{x}_{i}-{\mu }_{i}}{{\sigma }_{i}}$$

Where ${\mu }_{i}$ and ${\sigma }_{i}$ denote the mean and the standard deviation of the logit MFR of the $i$ th MCB across baseline samples.

Next, ${Z}_{i}$ was transformed into a *p*-value ${p}_{i}$, *P*-values of the multi-cancer markers were combined into a methylation score with Fisher's method.

2 $$\mathrm{Methylation}\;\mathrm{score}\;=\frac{-2\sum_{i=1}^I{\mathrm w}_i\ln p_i}{\sum_{i=1}^Iw_i}$$

Where ${w}_{i}$ is the coverage of the $i$th MCB.

### CSO marker selection

CSO markers were selected from cancer samples in the training cohort that were correctly identified as true positives by the diagnostic methylation score model with 98% specificity. Initially, 100 MCBs with the highest MI values were selected for each of the 21 (${C}_{7}^{2}$) pairwise comparisons among the seven cancer classes, resulting in a union of candidate CSO markers. Starting with the candidates, a multi-class logistic regression model with L2 penalty was fitted to 80% of the true-positive cancer samples from the training cohort. Markers were progressively removed from the model, one at a time, until the model reached the maximum Akaike Information Criterion (AIC). This backward elimination procedure was iterated 10 times with 10 bootstraps. The final CSO markers were MCBs retained in more than six bootstraps.

### CSO ensemble model

The MFR values of the CSO markers were used to construct a seven-class multi-layer stacked ensemble model using the AutoGluon machine learning framework [21] (Additional file 1: Fig. S1). Only the true positive cancer samples from the training cohort with 98% specificity were used to train the CSO ensemble model. Data preprocessing included logit transformation and Z-score normalization of the MFR values across samples for each marker, using the mean and standard deviation of the MFR values of the training samples. The ensemble model consisted of a base layer of various individual machine learning models whose predictions were aggregated with the preprocessed data to construct various stacker models in the first stacked layer. Weighted combinations of the first-layer stacker models were then aggregated to produce the final output probability of the ensemble model. Repeated k-fold bagging was used to tune the weights and the hyperparameters of the models to mitigate overfitting.

### Impact of the number of markers on multi-cancer diagnosis and CSO localization

Reducing the number of markers may lower the cost of the test and make it more affordable. To explore the possibility of making accurate prediction with fewer markers, we serially changed the number of markers in the diagnostic methylation score model and the CSO ensemble model, and evaluated the performance of the models constructed from the pruned markers. The calculation of the methylation score and the model fitting process of the CSO ensemble model remained the same as described in the *Diagnostic methylation score model* and the *CSO ensemble model* section, where the only thing changed was the input markers.

The multi-cancer diagnostic markers were eliminated from or appended to the original marker set according to MI calculated in the *Multi-cancer diagnostic marker selection*. Methylation scores were calculated for 100 times, using the union of MCBs with top 1, 2, 3, …, 100 MI for the seven cancer types as input features. The CSO markers were eliminated from or appended to the original marker set according to the 10 iterations of backward elimination in the *CSO marker selection*. We trained the CSO ensemble models for 10 times, using the markers being retained more than 0, 1, 2, …, and 9 times as input features.

## Results

### Methylation marker discovery for cancer diagnosis and cancer-signal-origin prediction

To construct a targeted methylation panel for multi-cancer detection and CSO localization, we selected DMRs covering a total of 15,589 hypermethylated CpGs in 32 TCGA solid tumor types to generate our custom 1-Mb hybridization capture panel. To reduce technical noise, we identified 6,042 MCBs covering 33,143 CpG sites and a total of 230 kb genomic regions for marker selection. Most of these MCBs were enriched in exons, introns, untranslated regions (UTRs), promoters, and enhancers, while only a few were located in repeat regions (Additional file 1: Methods, Fig. S2).

**Fig. 1:**
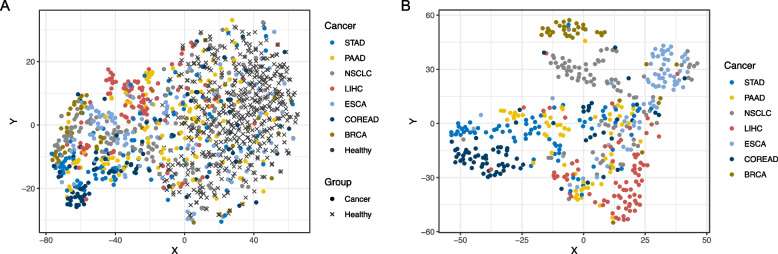
Visualization of the multi-cancer diagnostic markers and the CSO markers in cohort samples. t-SNE algorithm was used for dimension reduction. **A** Clustering of the 500 healthy controls and 733 cancer patients in the training and testing cohorts using 57 multi-cancer diagnostic markers. **B** Clustering of the 484 true positive cancer samples identified by the diagnostic methylation score model at 98% training specificity using 873 CSO markers

We used plasma samples from the training cohort to select MCB markers for multi-cancer diagnosis and CSO localization of the seven cancer types included in this study. For each cancer type, the top 10 MCBs with the highest MI with healthy controls were selected as multi-cancer diagnostic markers, yielding a total of 57 multi-cancer diagnostic MCBs across the seven cancer types. These markers were mapped to the exons of 18 genes and the promoters of 21 genes (Additional file 2: Table S3), among which five of the exon-overlapping genes (NSD1, ELMO1, ITGA4, HOXC13 and ITPKB) and three of the promoter-overlapping genes (ESRRG, MEF2D and PTPRU) were curated as cancer genes by the Network of Cancer Genes (NCG) database [22] (Additional file 2: Table S3). As expected, t-distributed stochastic neighbor embedding (t-SNE) plots showed apparent separation of cancer samples and healthy controls by the methylation profile of these 57 markers (Fig. 1A). Similarly, we performed CSO marker selection with pairwise MI among the seven cancer types. Using true positive cancer samples identified by methylation score with 98% training specificity, the top 100 markers with the highest MI for each cancer pair were initially selected. After backward elimination with 10 bootstraps, a set of 873 MCBs selected in more than six bootstraps were eventually retained as CSO markers. These markers were mapped to the exons of 218 genes and to the promoters of 299 genes (Additional file 2: Table S3), of which 40 and 42 were considered cancer relevant in the NCG database, respectively (Additional file 2: Table S4). In t-SNE analysis, cancer samples from different origins showed clear clustering using the methylation profile of these MCBs (Fig. 1B). Taken together, these data demonstrate the potential of our custom panel for multi-cancer detection and CSO localization.

### Performance of multi-cancer diagnosis and CSO localization

**Fig. 2:**
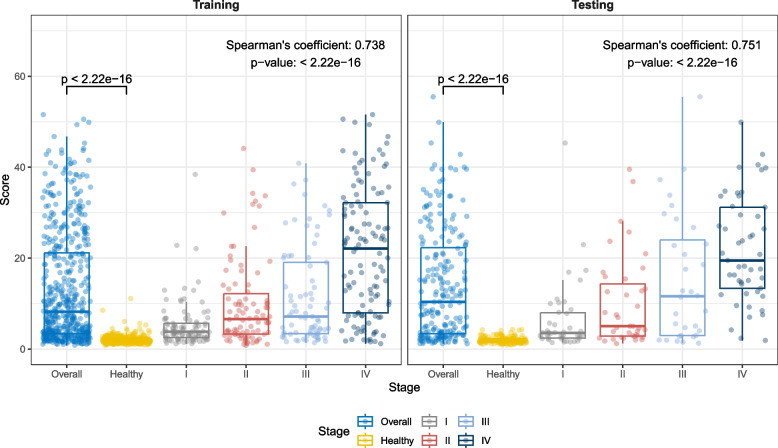
Methylation scores of healthy individuals and cancer patients stratified by stage. Samples in the training cohort and in the testing cohort were plotted separately. Two-sided Wilcoxon test was performed on the methylation score between healthy individuals and all cancer patients. Spearman's rank coefficient and one-sided p-value of correlation was calculated between the methylation score and the stage (i.e., healthy, stage I, II, III, and IV)

**Fig. 3:**
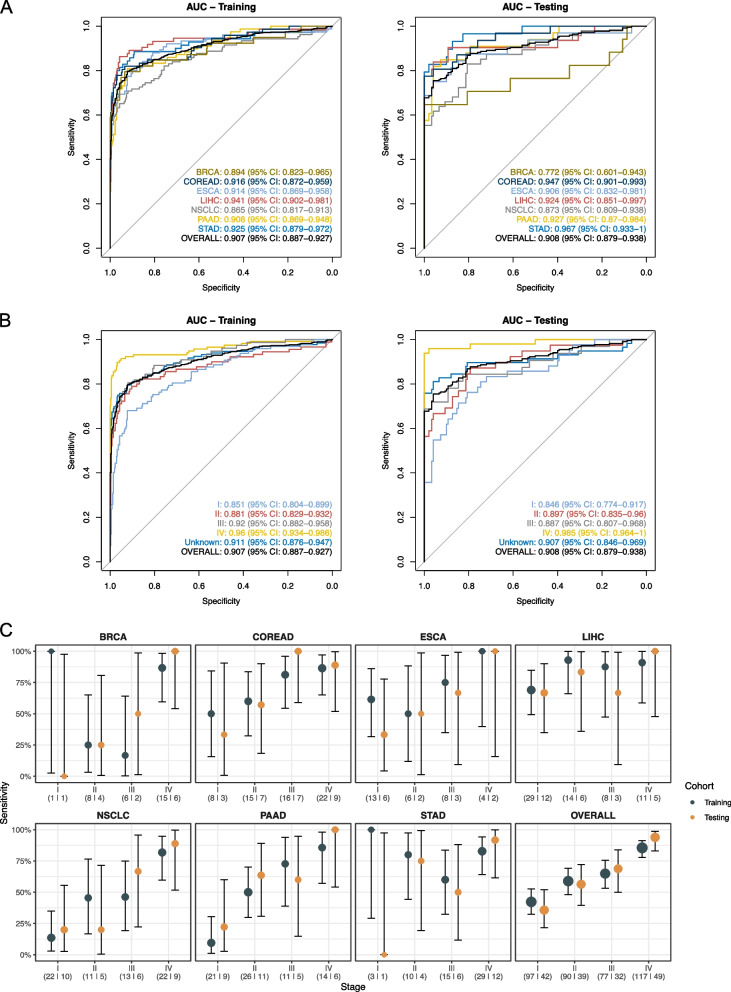
Performance of the diagnostic methylation score model. **A** RoC plots of the model, stratified by cancer type. **B** RoC plots of the model, stratified by stage. **C** Sensitivity of the model with 98% training specificity for individual cancer types or for multi-cancer, stratified by stage. Error bars indicate the 95% Wilson confidence interval (CI). The number of samples in the training and testing cohort is shown below the stage and separated by a vertical line

For a multi-cancer test to be clinically useful, we established a two-step approach including the diagnosis of cancer status and the localization of CSO after a positive cancer diagnosis. To profile the methylation pattern of a sample for cancer detection, the methylation levels of the 57 multi-cancer diagnostic MCB markers were quantified by MFR and we developed a metric called “methylation score” to summarize the deviations of MFR values of the 57 MCBs from the baseline distributions of training healthy controls. High methylation scores indicate abnormal methylation patterns, and as expected, we observed significantly higher methylation scores in cancer patients than in healthy individuals (Wilcoxon test *p*-value < 0.05) (Fig. 2). Using the diagnostic methylation score model to discriminate cancer samples from healthy controls, the overall AUC value was 0.907 (95% CI: 0.887–0.927) in the training cohort and 0.908 (95% CI: 0.879–0.938) in the testing cohort (Fig. 3A). With a cut-off value of 4.451 and a training specificity of 98% (i.e., the score threshold was set at the 98th quantile of the training control samples), the diagnostic methylation score model achieved a specificity of 100% (95% CI: 97.6%-100%) in the testing cohort. The overall sensitivity was 65.5% (95% CI: 61.2%-69.6%) and 67.3% (95% CI: 60.6%-73.4%) in the training and testing cohorts, respectively, and the sensitivity for early-stage (I and II) samples was 50.3% (95% CI: 37.7%-52.3%) and 45.7% (95% CI: 34.6%-57.1%), respectively (Fig. 3B). For most cancer types, detection sensitivity increased with increasing disease stage in both the training and testing cohorts (Fig. 3C). Spearman's rank coefficient of correlation between the methylation score and cancer stage was 0.738 and 0.751 in the training and testing cohort, respectively (Fig. 2), suggesting an association between methylation score and tumor load.

**Fig. 4:**
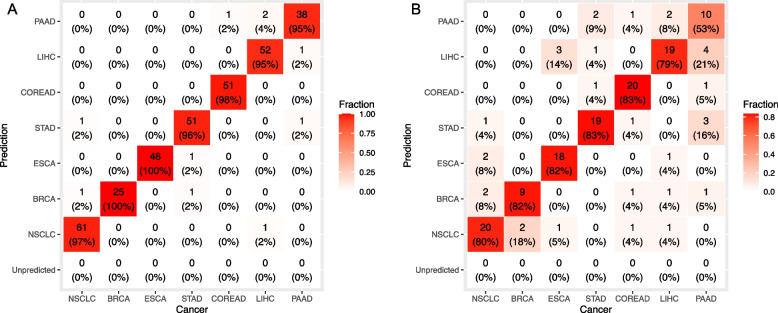
Performance of the CSO ensemble model. Heat maps showing the number and proportion of cancer samples classified into a given class in (**A**) the training cohort and (**B**) the testing cohort. Column labels represent actual sample classes and row labels represent predicted classes

For CSO localization, we used the MFR values of the 873 CSO markers to construct a seven-class multi-layer stacked ensemble model (the CSO ensemble model) using the AutoGluon machine learning framework [21]. We included only true positive cancer samples identified by methylation score with 98% specificity for model development (*n* = 336) and testing (*n* = 148). AutoGluon initially trained 13 models with the 873 methylation features and subsequently identified the optimal ensemble combination, consisting of 4 models (NeuralNetFastAI, LightGBMXT, LightGBM, and CatBoost), from this set. The input data and the code for the fitting process were available on Github (http://github.com/tchan1029/TOTEM). Performance of the CSO ensemble model was assessed by the top 1 accuracy (i.e., the true class matched the most likely class) and the top 2 accuracy (i.e., the true class matched the first or the second most likely class). The top 1 and top 2 accuracies were 97.0% (95% CI: 94.6%-98.6%) and 98.2% (95% CI: 96.2%-99.3%) in the training cohort and 77.7% (95% CI: 70.1%-84.1%) and 86.5% (95% CI: 79.9%-91.5%) in the testing cohort (Fig. 4). The top 1 and top 2 accuracies of the ensemble model in the testing cohort surpassed those of any individual models (Additional file 1: Table S2), demonstrating that the ensemble model leveraged the collective wisdom of different models, thereby reducing the risk of overfitting associated with any single individual model.

The purpose of the early detection approach introduced in this study is to guide, rather than replace, clinical diagnostic procedures. Therefore, even if the CSO misclassifies a case into a closely associated cancer type, such as liver and pancreatic cancer, which share similar diagnostic protocols (i.e., abdominal ultrasound and magnetic resonance imaging), it remains clinically valuable. Misclassifications between liver and pancreatic cancer can still benefit patients by excluding other low-probability cancers and directing subsequent medical investigations. To account for misclassified cases that could still benefit from the CSO model, we combined LIHC and PAAD into a single category, hepatopancreatic cancer (HPCA), and recalculated the probability of HPCA by summing up the probabilities of the two classes. Subsequently, the overall top 1 and top 2 accuracies for all cancers increased to 97.9% (95% CI: 95.8%-99.2%) and 98.8% (95% CI: 97.0%-99.7%), respectively, in the training cohort and to 82.4% (95% CI: 75.3%-88.2%) and 90.5% (95% CI: 84.6%-94.7%), respectively, in the testing cohort (Additional file 1: Fig. S3).

### Impact of the number of markers on multi-cancer diagnosis and CSO localization

To explore the possibility of making accurate prediction with fewer markers, we performed a serial elimination of markers and evaluated the performance of TOTEM constructed from the pruned markers. The AUCs of the diagnostic methylation score model for the detection across cancer types and stages remained stable when 21 markers (three markers per cancer type) or more were included. Even with a set of only seven markers (one per cancer type), the diagnostic model could still perform well with an overall AUC of 0.865 (95% CI: 0.841–0.889) and 0.866 (95% CI: 0.830–0.903) in the training and testing cohorts, respectively (Additional file 1: Fig. S4). After reducing the number of CSO markers from 873 to 214, which only included markers retained in all 10 bootstraps, the CSO ensemble model achieved an integrated top 1 accuracy of 64.9% (95% CI: 56.6%-72.5%) and an integrated top 2 accuracy of 83.1% (95% CI: 76.1%-88.8%) in the testing cohort (Additional file 1: Fig. S5). Despite the decrease in top 1 accuracy from 77.7% (95% CI: 70.1%—84.1%), the top 2 accuracy was consistent with the full set of markers. Samples from different groups were still clustered in the t-SNE plots (Additional file 1: Fig. S6). These results suggest that a subset of our methylation markers may suffice for accurate multi-cancer diagnosis and acceptable CSO localization.

### Independent validation of multi-cancer diagnosis and CSO localization

To further evaluate the generalizability of our approach, we recruited an independent validation cohort consisting of 143 healthy controls, 79 LIHC and 100 STAD patients based on availability. The diagnostic methylation score model and the CSO ensemble model that has been trained on the training cohort were applied to the validation samples. The MFR values of the 57 multi-cancer diagnostic markers and the 873 CSO markers in the training, testing and independent validation cohorts were summarized in Additional file 2: Table S5 and Table S6, respectively. The prediction values of the diagnostic methylation score model and the CSO ensemble model were summarized in Additional file 2: Table S7 and Table S8, respectively.

**Fig. 5:**
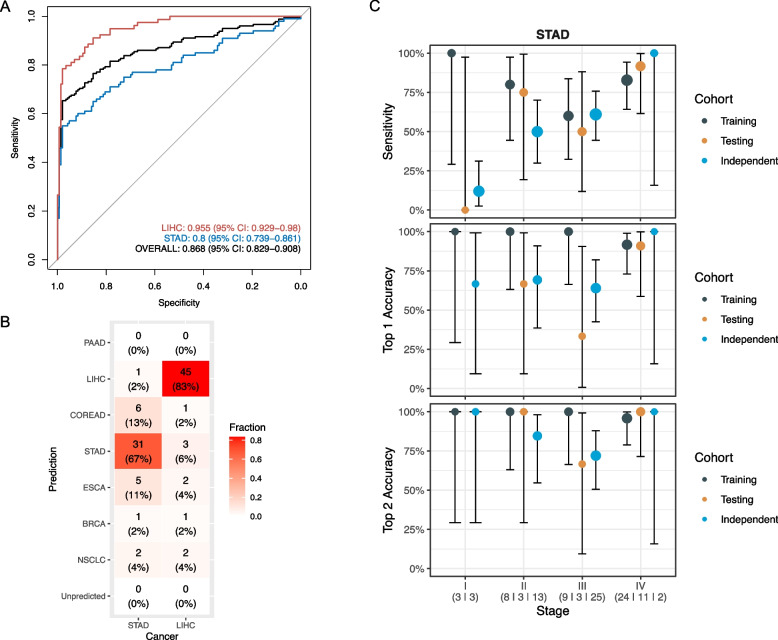
Performance of the diagnostic model and the CSO model in the independent validation cohort. **A** RoC curves of the diagnostic methylation score model; **B** Heat map showing the CSO prediction results for STAD validation samples; **C** Sensitivity, top 1 and top 2 accuracy of TOTEM for STAD validation samples at different stages compared with the training and testing cohorts. Error bars indicate the 95% Wilson CI

The AUC of the diagnostic model for the validation LIHC and STAD samples reached 0.955 (95% CI: 0.929–0.980) and 0.800 (95% CI: 0.739–0.861), respectively (Fig. 5A). Using the cutoff at 98% training specificity, the validated specificity was 98.6% (95% CI: 95.0%-99.8%) and the sensitivity was 68.4% (95% CI: 56.9%-78.4%) for LIHC and 46.0% (95% CI: 36.0%-56.3%) for STAD. With true positive samples at 98% training specificity, top 1 accuracy and top 2 accuracy of CSO localization were 83.3% (95% CI: 70.7%-92.1%) and 88.9% (95% CI: 77.4%-95.8%) for LIHC (*n* = 54), and 67.4% (95% CI: 52.0%-80.5%) and 78.3% (95% CI: 63.5%-89.1%) for STAD (*n* = 46), respectively (Fig. 5B). While the model performance for the LIHC validation samples was similar to the testing cohort, there was a decrease in the performance for the STAD validation samples, likely due to the lower proportion of stage IV cases. Indeed, when STAD samples were stratified by stage, we found no significant difference in the performance of diagnosis and CSO localization between the testing cohort and the validation cohort (Fig. 5C). Overall, the performances of the diagnostic methylation score model and the CSO ensemble model in LIHC and STAD patients within the independent validation cohort were comparable to those in the testing cohort, suggesting that our approach could be generalized to external data, specifically in these cancer types.

## Discussion

Utilizing the methylation profile of circulating cfDNA for non-invasive early cancer diagnosis has shown great promise, but currently there are only limited published large-scale investigations for multi-cancer application. In this study, we present a novel multi-cancer detection and localization approach based on targeted methylation sequencing of 1,555 plasma samples, which achieved high sensitivity and accurate CSO prediction for seven common cancers across stages. In particular, we explored the minimal number of markers required and demonstrated that comparable performance can be achieved with as few as 21 diagnostic markers and 214 CSO markers, achieving a training AUC of 0.865, a testing AUC of 0.866, and an integrated top 2 accuracy of 83.1% in the testing cohort.

Overfitting is a common concern in the field of early cancer detection due to the extremely low presence of tumor-derived cfDNA fragments. Unlike most published studies, we used a fragment-level metric rather than machine learning method for multi-cancer diagnosis, which likely mitigated the risk of overfitting problem. Indeed, we observed a consistent distribution of methylation scores between the training and testing cohorts across cancer class or stage (Additional file 1: Fig. S7). We also observed a strong association between methylation score and disease stage, with higher methylation scores generally associated with later stages, suggesting that we detected bona fide cancer-related methylation signals. Finally, to assess the potential bias of our method, we included an independent validation cohort of LIHC and STAD samples, which showed similar detection performance to both the training and testing cohorts when stratified by stage. Differences in the mean age between healthy individuals and patients were observed in the independent validation cohort (39.5 vs. 58.5 yrs). To validate the independence of methylation scores from age, individuals in the independent validation cohort were categorized into three groups: healthy individuals, LIHC patients, and STAD patients and further subdivided into younger (≤ 60 yrs) and older (> 60 yrs) subgroups. Wilcoxon tests were then conducted on the methylation scores of the younger and older subgroups within each group. The p-values from the three sets of tests were all non-significant (0.15, 0.67, and 0.14), indicating that methylation scores are not associated with age (Additional file 1: Fig. S8). Additionally, although the mean age of healthy individuals in the independent validation cohort (39.5 yrs) was different from that in the training (52.0 yrs) and testing cohort (51.5 yrs), no significant difference was found in the methylation scores of healthy individuals between the three cohorts (Additional file 1: Fig. S7). This suggests that the methylation score of healthy individuals was not associated with age. Taken together, these data demonstrate the robustness of our approach.

In contrast to the diagnostic model for multi-cancer binary classification, machine learning algorithms were implemented for the multi-class CSO prediction. Due to tumor heterogeneity, it was challenging to find a generic set of CSO markers applicable to all patients across the seven cancer classes, and thus the number of CSO markers was large relative to the sample size. We took several measures to reduce overfitting, including bootstrapping CSO marker selection, repeated bagging for hyperparameter tuning, and integrating predictions from multiple classifiers. Although there was still a decrease in CSO accuracy from the training to the testing cohort, similar performance was achieved in the independent validation dataset (top 1 accuracy: 76.0%; top 2 accuracy: 84.0%) compared to the testing cohort (top 1 accuracy: 77.7%; top 2 accuracy: 86.5%), suggesting that our CSO model can still generalize to external data with acceptable accuracy. In addition, we examined the association between cancer signal intensity and CSO accuracy and found that samples with higher methylation scores were more likely to be accurately predicted by the CSO model (Additional file 1: Fig. S9). For example, for samples with methylation scores above 7.36, which accounted for 80.1%, 86.5%, and 66% of the true positives in the training, testing and the independent datasets, the top 1 accuracy increased to 98.5%, 80.5%, and 80.3%, and the top 2 accuracy increased to 98.9%, 89.8%, and 89.4%, respectively. In comparison, for samples with methylation scores above 4.451 (i.e., true positives with the cutoff providing 98% training specificity), the top 1 accuracy was 97.0%, 77.7%, and 76.0%, and the top 2 accuracy was 98.2%, 86.5%, and 84.0%, respectively. This result suggests that our CSO model was built on true tumor-related methylation signals instead of technical or biological noise.

Methylation-based multi-cancer detection usually requires a large number of markers due to cancer heterogeneity. For example, Liu et al. selected 256 features from a 17.2 Mb panel for each ordered cancer type pair and concatenated them to train a multi-class model in more than 50 cancer types [3]. Gao et al. selected 566 pan-cancer and 1,240 tissue-specific methylation regions from a 2.7 Mb panel and performed a multi-class classification in six cancer types (COREAD, ESCA, LIHC, NSCLC, OV, PAAD) [13]. Compared with these published multi-cancer studies with larger panels, our approach could maintain comparable sensitivities with only 57 markers for cancer detection in both stage I (Liu et al.: 39.0%; Guo et al.: 35.4%; TOTEM: 35.7%) and stage II (Liu et al.: 69.0%; Guo et al.: 54.5%; TOTEM: 56.4%) and achieve comparable top 1 accuracy for the five overlapping cancer types (Liu et al.: 93.0%; Guo et al.: 80.8%; TOTEM: 76.3%) with 873 markers for CSO identification. We also investigated the influence of the number of markers on the performance of cancer diagnosis and localization for TOTEM. Satisfactory performance could be achieved with only 21 diagnostic markers and 214 CSO markers. This tremendous reduction in the size of the marker set could significantly reduce the cost of panel design and NGS sequencing. It also opens up the possibility of developing a multiplex PCR-based platform to further reduce the cost of testing and simplify the experimental workflow, which would benefit the applicability of our approach for large-scale cancer screening purposes. Moreover, the integration of protein and methylation cfDNA markers has been shown to enhance the discrimination for early-stage cancer detection, as shown by Ben-Ami et al. in a pancreatic cancer cohort [23]. This combination strategy holds promise for improving the sensitivity of multi-cancer diagnosis, particularly in the early stages.

In addition to targeted methylation sequencing studies, there are also studies utilizing shallow whole-genome methylation sequencing for cancer diagnosis and cancer signal origin prediction. Comparing with targeted approach, which only analyzes differentially hypermethylated or hypomethylated CpG sites enriched in CpG islands, the whole-genome approach profiles whole-genome hypomethylation and allows for multimodal analysis of methylation and fragmentation, therefore suffers less from tumor heterogeneity, and could contribute to higher sensitivity in pan-cancer diagnosis. However, high-depth targeted sequencing usually has higher analytical sensitivity in detecting cancer-specific genetic changes [24]. The low coverage of whole genome methylation sequencing poses a challenge in capturing cancer-specific signals. THEMIS, a multimodal analysis integrating methylation, fragment size, fragment end motif, and copy number changes from enzymatic conversion-based whole-genome methylation sequencing data, demonstrated superior sensitivity in detecting early-stage patients (73% and 74% at 99% specificity in the training and testing cohorts, respectively). Nevertheless, this enhanced sensitivity came at the expense of compromised CSO accuracy. The CSO accuracy of THEMIS approach was only 53% and 54% in the training and testing datasets, respectively [25], which was lower than the top 1 accuracy observed in the TOTEM study.

The present study has several limitations that need to be addressed in the future. First, only healthy individuals were included in the study as controls, and therefore the performance of our classifiers was not evaluated for the high-risk population with cancer-related benign conditions. Second, the independent validation cohort recruited only LIHC and STAD patients based on availability, thus the performance for the other five cancer types remains to be externally validated. Finally, the non-cancer status of the healthy controls was determined at the time of recruitment without follow-up, which may misclassify early-stage cancers as controls and overestimate the false positive rate. The real-world clinical performance of our approach needs to be investigated in a much larger prospective cohort, including all seven cancer types and balanced disease stages. As more cancer subtypes and samples are included in the study, the tumor diversity within the cohort will be enriched. This will enable us to identify more diverse and effective markers, thereby enhancing the robustness of the model. Complete long-term follow-up of non-cancer controls is also necessary to evaluate performance.

## Conclusion

The diagnostic model achieved an overall area under the curve (AUC) of 0.907 and 0.908 in the training and testing cohorts, respectively. For cancer patients correctly identified by the diagnostic model in the testing cohort, the top 1 and top 2 CSO accuracies were 77.7% and 86.5%, respectively. Notably, performance was maintained with only 21 diagnostic and 214 CSO markers, achieving a training AUC of 0.865, a testing AUC of 0.866, and an integrated top 2 accuracy of 83.1% in the testing cohort. These results demonstrate the promising potential of our approach for accurate multi-cancer detection and localization by plasma methylation profiling. The real-world clinical performance of our approach needs to be investigated in a much larger prospective cohort.

### Supplementary Information

Supplementary Material 1.

Supplementary Material 2.

## Data Availability

The datasets analysed during the current study are available in the the Genome Sequence Archive for Human (https://ngdc.cncb.ac.cn/gsa-human/) with the accession number HRA005803.

## Abbreviations

AIC: Akaike Information Criterion
AUC: Area under the curve
BRCA: Breast cancer
cfDNA: Cell-free DNA
CI: Confidence interval
COREAD: Colorectal cancer
CSO: Cancer signal origin
ctDNA: Circulating tumor DNA
DMP: Differentially methylated position
DMR: Differentially methylated region
EM-seq: Enzymatic methyl sequencing
ESCA: Esophageal cancer
FDR: False discovery rate
HPCA: Hepatopancreatic cancer
LIHC: Liver cancer
MCB: Methylation-correlated block
MFC: The count of methylated fragments
MFR: The ratio of fully methylated fragments
MI: Mutual information
NCG: The Network of Cancer Genes
NSCLC: Non-small cell lung cancer
PAAD: Pancreatic cancer
RoC: Receiver Operating Characteristic
STAD: Stomach cancer
t-SNE: T-distributed stochastic neighbor embedding
TCGA: The Cancer Genome Atlas
TFBS: Transcription factor binding site
TOTEM: CTdna Origin Tracker dependent on Epigenetic Methylation markers
TSS: Transcription start site
UTR: Untranslated region
WGBS: Whole-genome bisulfite sequencing
