# Supplementary material for: TOTEM: a multi-cancer detection and localization approach using circulating tumor DNA methylation markers
Source: BMC Cancer. 2024 Jul 15;24:840. doi: 10.1186/s12885-024-12626-7 (PMC11247868; doi:10.1186/s12885-024-12626-7)
Supplement: Supplementary file 1 — Supplementary Material 1. [file 12885_2024_12626_MOESM1_ESM.docx]

**Supplementary Methods**

***Design of methylation panel***

The methylation panel for targeted sequencing was designed to cover cancer-specific hypermethylated DMRs merged from differentially methylated positions (DMPs) selected from publicly available 450k array data.

By comparing 8,268 primary tumors and 734 normal adjacent tissues from the TCGA database, CpG sites were selected as tumor-versus-normal hypermethylated DMPs for each cancer type if: (1) the false discovery rate (FDR) of the empirical Bayes moderated t-test p-value calculated by the R package limma (1) is below 0.05; (2) mean(beta-value) + SD(beta-value) < 0.1 in normal tissue; (3) mean(beta value) ≥ 0.3 in tumor; (4) distance to the nearest CpG site ≤ 250 bp.

Similarly, By comparing primary tumors from the TCGA database and 656 peripheral blood samples from healthy individuals from GSE40279 (2), CpG sites were selected as tumor-versus-WBC hypermethylated DMPs for each cancer type if: (1) Limma (1) test FDR < 0.05; (2) mean(beta-value) + SD(beta value) < 0.1 in blood; (3) mean(beta-value) ranked top 5000 in tumor; (4) distance to the nearest CpG site ≤ 250 bp.

In total, 8,975 tumor-versus-normal DMPs and 8,294 tumor-versus-blood hypermethylated DMPs were obtained for 32 solid tumor types. DMPs within 250 bp were then merged into DMRs.

***Data preprocessing***

Methylation sequencing reads were demultiplexed by Illumina bcl2fastq. Adaptors were trimmed by Trimmomatic (v.0.36). Reads were aligned against the human reference genome (hg19) and de-duplicated by BisMark (v0.19.0). Samtools (v.1.3) and BamUtil (1.0.14) were used for sorting and overlap-clipping of mapped reads. Reads with mapping quality below 20 or conversion rate of non-CpG cytosines below 95% were filtered out. After preprocessing, the average depth exceeded 1000x in half of the cfDNA samples.

***Annotation of MCBs***

Annotations of exons, introns, 5’ UTRs, 3’ UTRs, CpG islands and repeat regions were downloaded from the UCSC Genome Browser database (3).Promoters were defined as the regions from 4 kb upstream of transcription start sites (TSSs) to 1 kb downstream of TSSs. Genomic coordinates of enhancers and transcription factor binding sites (TFBSs) were downloaded from the EnhancerDB database (4).

***Mutual Information calculation***

To compare the difference in methylation patterns between two groups, the MFC value was converted into a binary factor $m_{c}\in\{0,1\}$, where 0 indicated an MFC below a given cutoff value $c$ and 1 indicated an MFC greater than or equal to $c$.

The diagnostic markers were selected by calculating the cancer-specific MI, denoted as $I(M_{c};Y)$, between $m_{c}$ and the outcome $Y=\{H,A\}$.

$I(M_{c};Y)=\sum_{y\in Y} \sum_{m_{c}\in\{0,1\}} P(m_{c},y)\log_{2} \frac{P(m_{c},y)}{P(m_{c})p(y)}$ (1.1)

where $H$ denotes healthy individuals and $A$ denotes patients with cancer type A.

If $m_{c}=0$ was assumed to provide no information, Equation (1.1) could be simplified to

$I(M_{c};Y)\approx\sum_{y\in Y} P(m_{c}=1,y)\log_{2} \frac{P(m_{c}=1,y)}{P(m_{c}=1)P(y)}$ (1.2)

Using the weak prior $P(H)=P(A)=0.5$, we had

$P(m_{c}=1)=\sum_{y\in Y} P(m_{c}=1|y)P(y)=\frac{P(m_{c}=1|H)+P(m_{c}=1|A)}{2}$ (1.3)

According to Bayes’ theorem and Equation (1.3), Equation (1.2) could be transformed into

$I(M_{c};Y)\approx\frac{P(m_{c}=1|H)}{2}\log_{2} \frac{2\cdot P(m_{c}=1|H)}{P(m_{c}=1|H)+P(m_{c}=1|A)}+\frac{P(m_{c}=1|A)}{2}\log_{2} \frac{2\cdot P(m_{c}=1|A)}{P(m_{c}=1|H)+P(m_{c}=1|A)}$ (1.4)

where $P(m_{c}=1|H)$ and $P(m_{c}=1|A)$ are the probability of observing $m_{c}=1$ in a healthy individual and the probability of observing $m_{c}=1$ in a patient with cancer type A, respectively.

Specifically, $P(m_{c}=1|H)$ was estimated directly from $f_{H}$, where $f_{H}$ is the proportion of healthy individuals with $m_{c}=1$. Given that the plasma of cancer patients consists of both ctDNA derived from tumor cells and cfDNA derived from normal cells, the probability of observing the same event in a patient with type A cancer was estimated from both $f_{H}$ and $f_{A}$, where $f_{A}$ is the proportion of type A cancer patients with $m_{c}=1$.

$P(m_{c}=1|A)=f_{H}+(1-f_{H})f_{A}$ (1.5)

For each candidate MCB, the cutoff $c$ was optimized by a grid search to find the maximum $I(M_{c};Y)$, where c is an integer ranging from 1 to 20.

The selection of CSO markers was similar to the selection of diagnostic markers, except that the outcome became $Y$=$\{A,B\}$, denoting the pairwise comparison between cancer types A and B.

**Supplementary Data**

**Table S1 Demographics and clinical information of participants enrolled in this study**

|  | **Training Set** | | **Testing Set** | | **Independent Valiation Set** | |
| --- | --- | --- | --- | --- | --- | --- |
|  | **Case** | **Control** | **Case** | **Control** | **Case** | **Control** |
| **Total, n (%)** | 513 (59.4%) | 350 (40.6%) | 220 (59.5%) | 150 (40.5%) | 179 (55.6%) | 143 (44.4%) |
| **Age, years (%)** |  |  |  |  |  |  |
| mean±SD | 59.8±11.2 | 52.0±9.2 | 61.9±11.6 | 51.5±8.6 | 58.9±10.4 | 39.5±10.4 |
| 19-59 | 240 (46.8%) | 280 (80%) | 91 (41.4%) | 127 (84.7%) | 88 (49.2%) | 134 (93.7%) |
| 60-92 | 273 (53.2%) | 69 (19.7%) | 128 (58.2%) | 23 (15.3%) | 91 (50.8%) | 8 (5.6%) |
| NA | 0 | 1 (0.3%) | 1 (0.5%) | 0 | 0 | 1 (0.7%) |
| **Gender (%)** |  |  |  |  |  |  |
| male | 300 (58.5%) | 173 (49.4%) | 138 (62.7%) | 74 (49.3%) | 137 (76.5%) | 73 (51.0%) |
| female | 213 (41.5%) | 177 (50.6%) | 82 (37.3%) | 76 (50.7%) | 42 (23.5%) | 70 (49.0%) |
| **Stage (%)** |  |  |  |  |  |  |
| I | 97 (18.9%) |  | 42 (19.1%) |  | 25 (14.0%) |  |
| II | 90 (17.5%) |  | 39 (17.7%) |  | 26 (14.5%) |  |
| III | 77 (15.0%) |  | 32 (14.5%) |  | 41 (22.9%) |  |
| Ⅳ | 117 (22.8%) |  | 49 (22.3%) |  | 2 (1.1%) |  |
| NA | 132 (25.7%) |  | 58 (26.4%) |  | 85 (47.5%) |  |
| **Diagnosis (%)** |  |  |  |  |  |  |
| Healthy |  | 350 (40.6%) |  | 150 (40.5%) |  | 143 (44.4%) |
| Breast | 39 (7.6%) |  | 17 (7.7%) |  |  |  |
| Colorectal | 72 (14.0%) |  | 31 (14.1%) |  |  |  |
| Esophageal | 75 (14.6%) |  | 32 (14.5%) |  |  |  |
| Gastric | 70 (13.6%) |  | 29 (13.2%) |  | 100 (55.9%) |  |
| Liver | 73 (14.2%) |  | 31 (14.1%) |  | 79 (44.1%) |  |
| Lung | 106 (20.7%) |  | 47 (21.4%) |  |  |  |
| Pancreatic | 78 (15.2%) |  | 33 (15.0%) |  |  |  |

Statistics on the age and gender of all participants, and the stage and cancer type distributions of cancer patients in the training, testing and independent validation sets.

**Table S2 CSO Performance of the individual models and the ensemble model**

| **Model** | **Top 1 Accuracy** | | | **Top 2 Accuracy** | | |
| --- | --- | --- | --- | --- | --- | --- |
|  | **Training** | **Testing** | **Independent Validation** | **Training** | **Testing** | **Independent Validation** |
| CatBoost | 96.1% | 74.3% | 65.0% | 97.6% | 82.4% | 81.0% |
| ExtraTreesEntr | 94.3% | 71.6% | 54.0% | 97.3% | 80.4% | 68.0% |
| ExtraTreesGini | 94.6% | 69.6% | 63.0% | 97.6% | 80.4% | 78.0% |
| KNeighborsDist | 93.2% | 58.8% | 50.0% | 96.1% | 73.6% | 58.0% |
| KNeighborsUnif | 82.1% | 64.9% | 56.0% | 97.3% | 83.1% | 80.0% |
| LightGBM | 95.8% | 74.3% | 60.0% | 98.5% | 83.8% | 72.0% |
| LightGBMLarge | 94.6% | 64.9% | 41.0% | 97.3% | 77.7% | 60.0% |
| LightGBMXT | 96.1% | 77.0% | 64.0% | 98.2% | 83.1% | 76.0% |
| NeuralNetFastAI | 96.7% | 76.4% | 73.0% | 98.5% | 85.1% | 84.0% |
| NeuralNetTorch | 94.9% | 77.0% | 71.0% | 97.9% | 85.1% | 87.0% |
| RandomForestEntr | 96.1% | 73.0% | 63.0% | 97.3% | 82.4% | 77.0% |
| RandomForestGini | 95.8% | 72.3% | 51.0% | 97.3% | 83.8% | 66.0% |
| WeightedEnsemble_L2 | 97.0% | 77.7% | 76.0% | 98.2% | 86.5% | 84.0% |


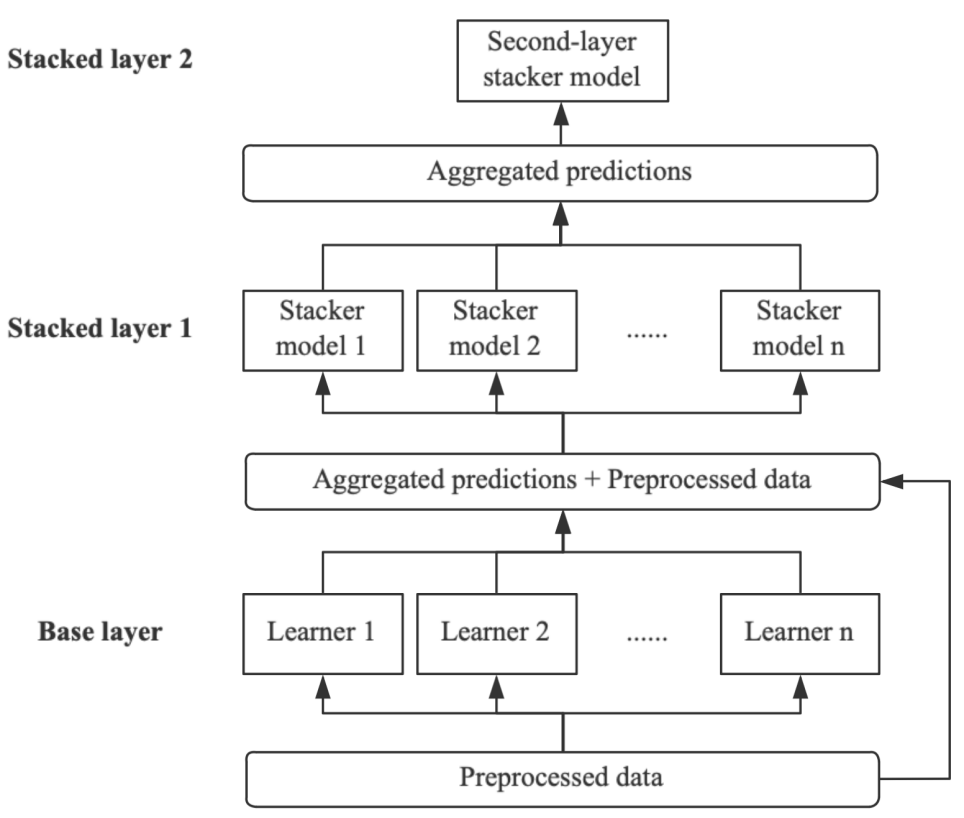


**Fig. S1** **Illustration of the CSO multi-layer stacked ensemble model.** The multi-layer stacked ensemble model consisted of a base layer and two stacked layers: (1) Base layer: base learners such as boosted trees and neural network models using the preprocessed CSO markers as inputs; (2) Stacked layer 1: stacker models using the aggregated predictions of the “base models” and the preprocessed CSO markers as inputs; (3) Stacked layer 2: second-layer stacker model using the prediction of the “stacker models” as inputs.


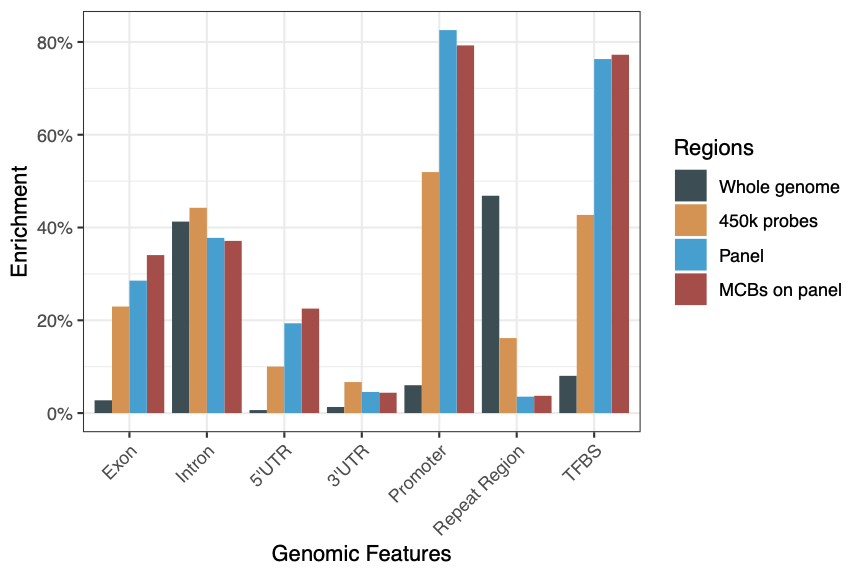


**Fig. S2 Genomic features of the 6,042 MCBs identified.** The bar graph shows the percentage of bases in the whole genome, the 450k array probes, the 1Mb panel regions or the MCBs that overlapped with the genomic features.

| A.  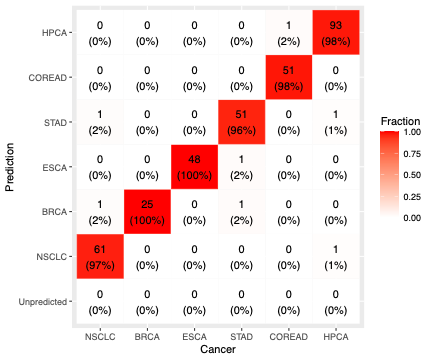 | B.  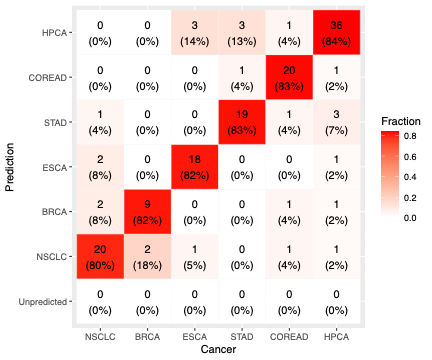 |
| --- | --- |

**Fig. S3. Performance of the CSO ensemble model after merging PAAD and LIHC into one class (HPCA).** Heat maps showing the number and proportion of cancer samples classified to a given class in (A) the training cohort and (B) the testing cohort. Column labels represent actual sample classes and row labels represent predicted classes.

A.


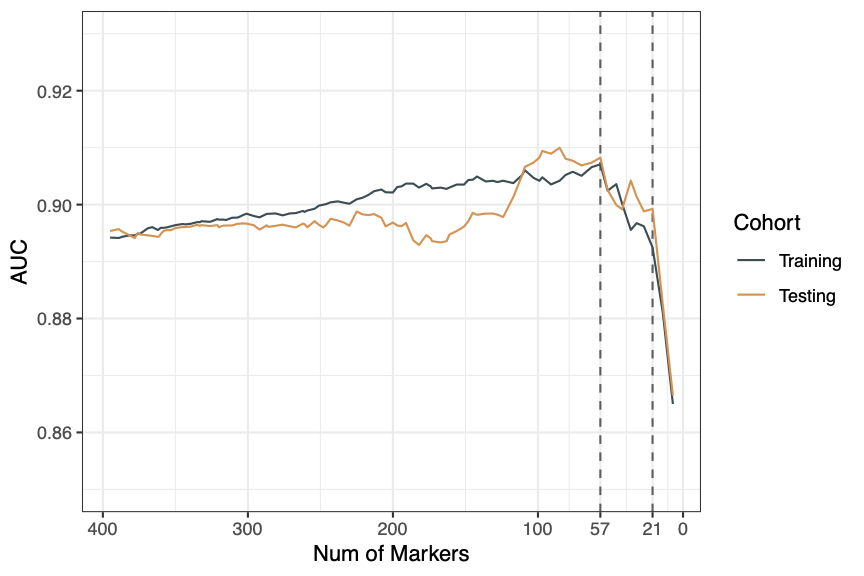


B.


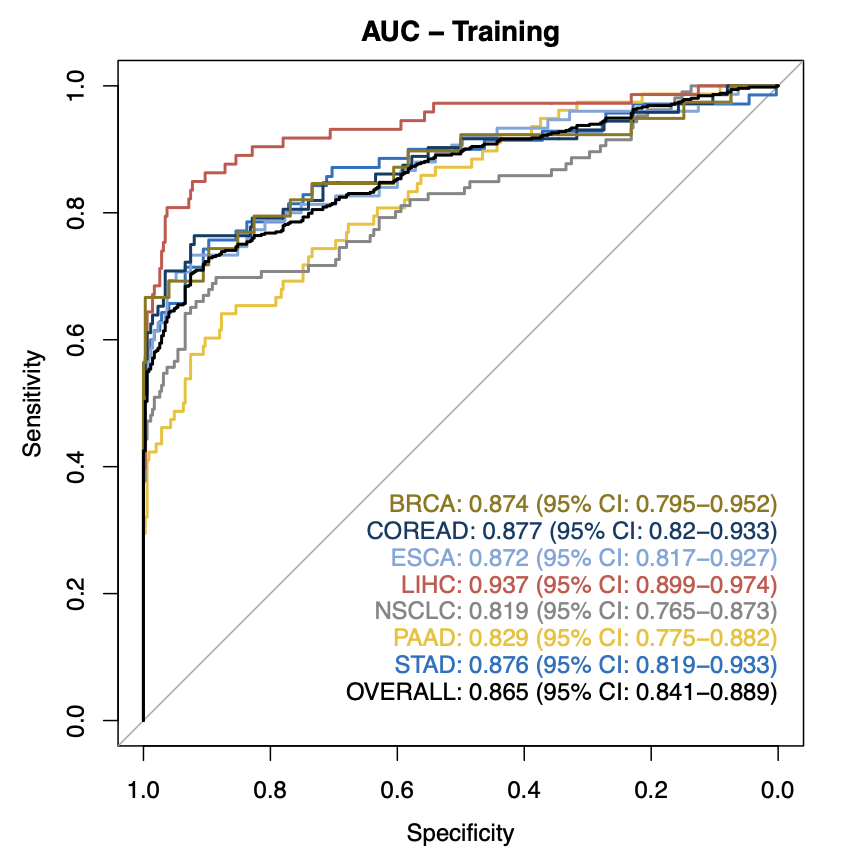

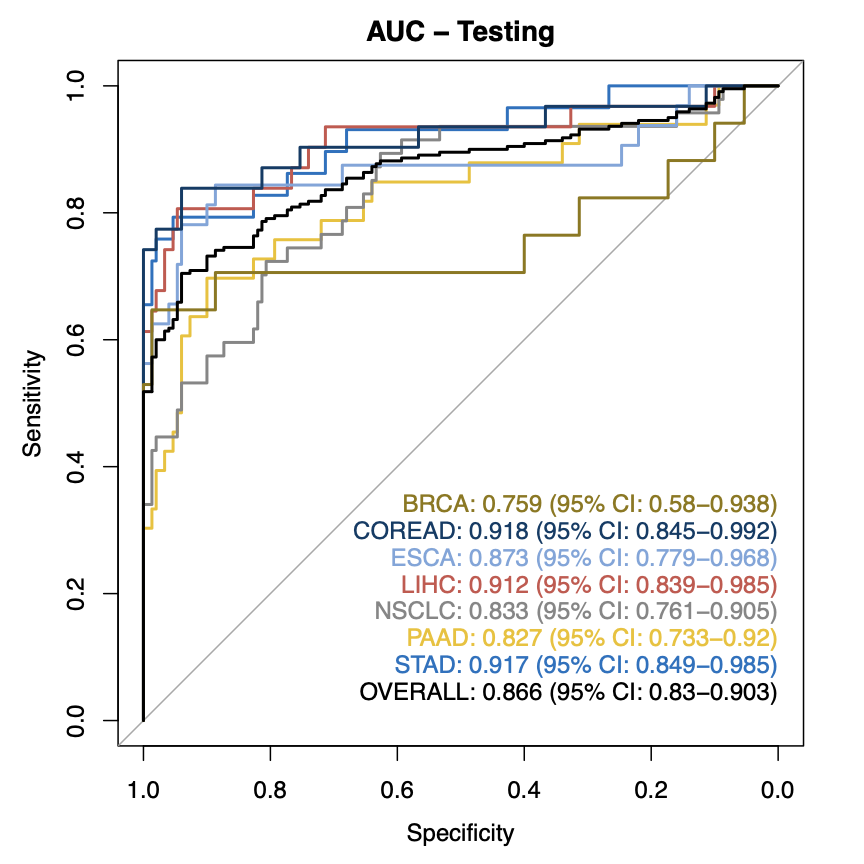


**Fig. S4 AUC values of the diagnostic methylation score models constructed with different numbers of markers.** (A) Line graphs show the change in performance, with the number of markers on the x-axis and the diagnostic AUC on the y-axis; (B) RoC curves of the updated model built with a reduced marker set of 7 markers.

A.


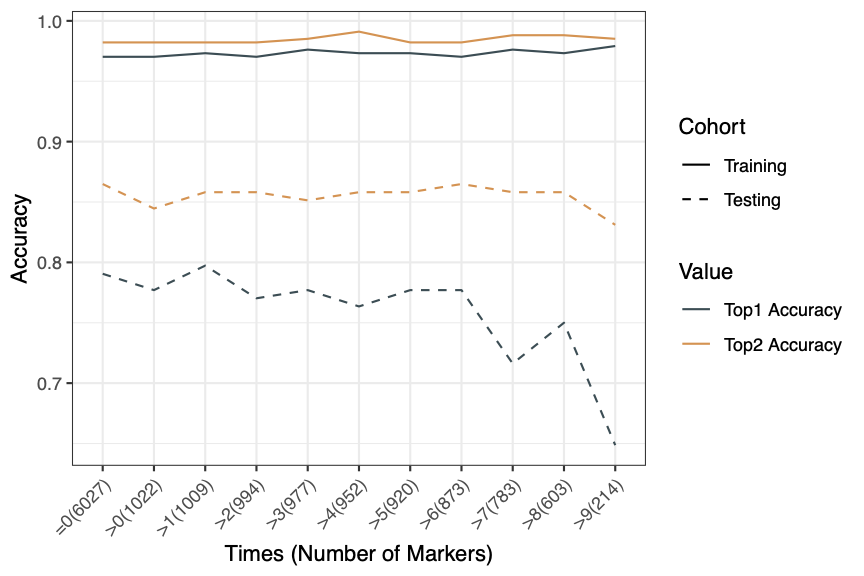


B.


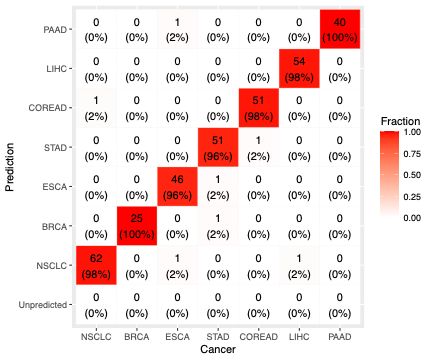

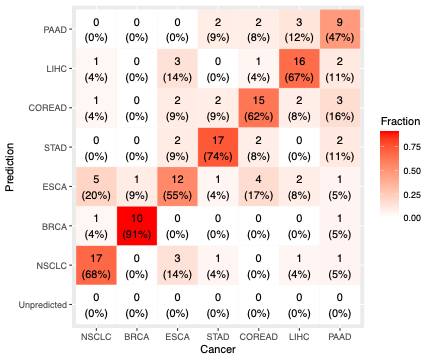


**Fig. S5 Accuracies of the CSO ensemble models constructed with different numbers of markers.** (A) Line plots show the change in performance, with the number of markers on the x-axis and the top 1 and top 2 accuracies on the y-axis; (B) Heat maps show the numbers and proportions of cancer samples classified into a given class in the training and test cohorts of the updated model built with a reduced marker set of 214 markers, with the actual sample classes on the column labels and the predicted classes on the row labels.

| A.   | B.  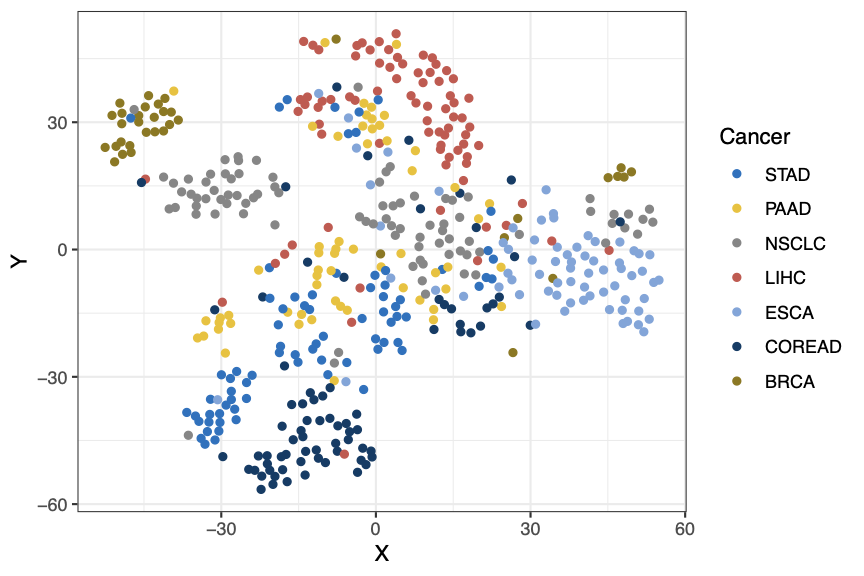 |
| --- | --- |

**Fig. S6 Visualization of the pruned multi-cancer diagnostic markers and the pruned CSO markers in the cohort.** (A) Clustering of the 500 healthy controls and 733 cancer patients in the training and testing cohort using 7 diagnostic markers. (B) Clustering of the 484 true positive cancer samples identified by the diagnostic methylation score model with 98% training specificity using 214 CSO markers. t-SNE algorithm was used for dimension reduction.

| A.  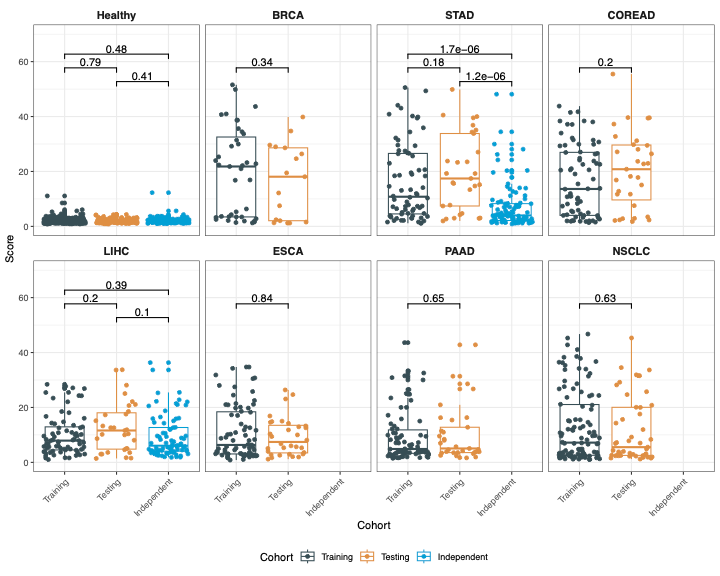 | B.  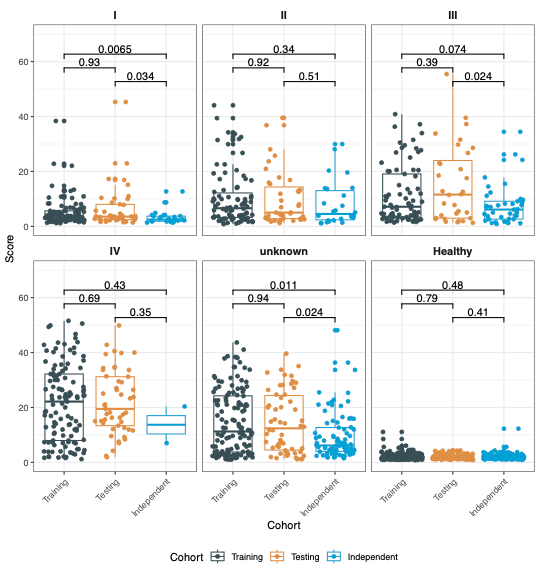 |
| --- | --- |

**Fig. S7 Methylation scores of healthy controls and cancer samples.** Methylation scores for all samples in the training, testing, and validation cohorts were plotted as boxplots stratified by (A) cancer class or (B) clinical stage. Two-sided Wilcoxon tests were performed between each pair.

**Fig. S8 Methylation scores of younger and older subgroups in the independent validation cohort.** Methylation scores for all samples in the validation cohorts were plotted as boxplots stratified by age and cancer class. Two-sided Wilcoxon tests were performed between the younger (≤60 yrs) and older (>60 yrs) group in each cancer class.


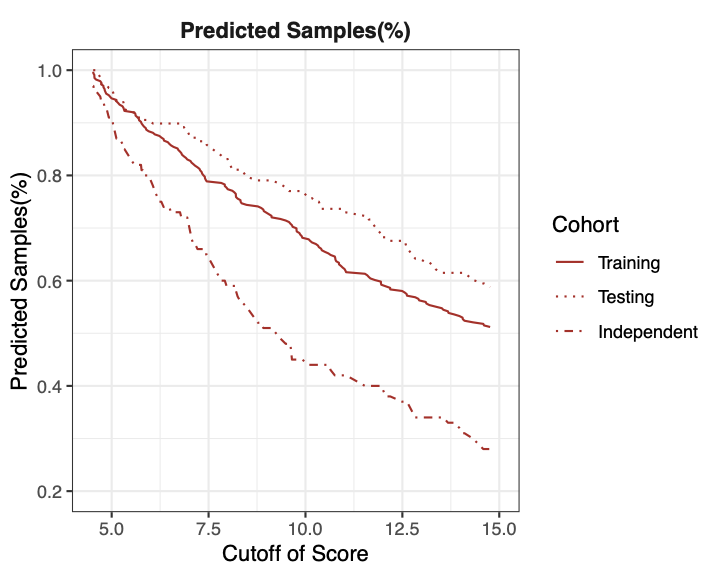

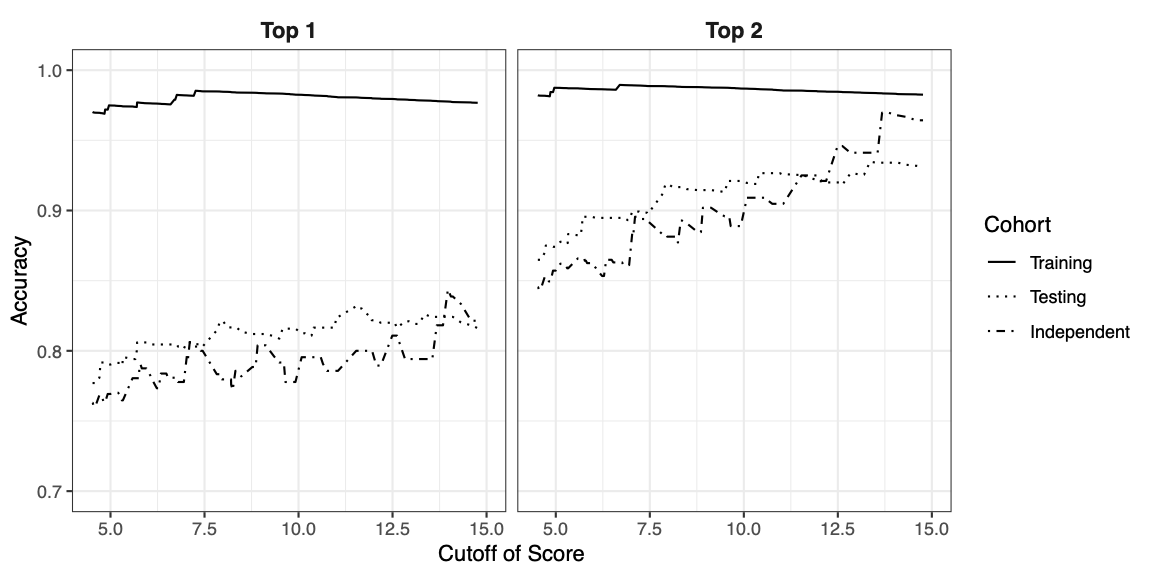


**Fig. S9 Performance of the CSO model with cancer samples subset by methylation score thresholds.** Line graph with the methylation score threshold on the x-axis and the percentage of samples meeting the score requirement or the accuracy on the y-axis. Red lines represent the percentage of samples with a score above the threshold. Black lines represent the accuracy of these samples. Line shapes annotate samples from different cohorts.

**References**

1. Ritchie ME, Phipson B, Wu D, Hu Y, Law CW, Shi W, et al. limma powers differential expression analyses for RNA-sequencing and microarray studies. Nucleic Acids Res. 2015 Apr 20;43(7):e47.

2. Hannum G, Guinney J, Zhao L, Zhang L, Hughes G, Sadda S, et al. Genome-wide methylation profiles reveal quantitative views of human aging rates. Mol Cell. 2013 Jan 24;49(2):359–67.

3. Kent WJ, Sugnet CW, Furey TS, Roskin KM, Pringle TH, Zahler AM, et al. The human genome browser at UCSC. Genome Res. 2002 Jun;12(6):996–1006.

4. Kang R, Zhang Y, Huang Q, Meng J, Ding R, Chang Y, et al. EnhancerDB: a resource of transcriptional regulation in the context of enhancers. Database. 2019 Jan 1;2019:bay141.
